# Identification of Tick-Borne Pathogens and Genotyping of *Coxiella burnetii* in *Rhipicephalus microplus* in Yunnan Province, China

**DOI:** 10.3389/fmicb.2021.736484

**Published:** 2021-09-21

**Authors:** Jun Jiao, Jianing Zhang, Peisheng He, Xuan OuYang, Yonghui Yu, Bohai Wen, Yi Sun, Qinghong Yuan, Xiaolu Xiong

**Affiliations:** ^1^State Key Laboratory of Pathogen and Biosecurity, Beijing Institute of Microbiology and Epidemiology, Beijing, China; ^2^Yunnan Provincial Key Laboratory of Natural Focal Disease Control and Prevention, Yunnan Institute of Endemic Diseases Control and Prevention, Dali, China

**Keywords:** *Rhipicephalus microplus*, *Candidatus Rickettsia jingxinensis*, *Anaplasma marginale*, *Coxiella burnetii*, Coxiella-like endosymbiont, MLVA, Yunnan province

## Abstract

*Rhipicephalus microplus*, a vector that can transmit many pathogens to humans and domestic animals, is widely distributed in Yunnan province, China. However, few reports on the prevalence of tick-borne pathogens (TBPs) in *Rh. microplus* in Yunnan are available. The aim of this study was to detect TBPs in *Rh. microplus* in Yunnan and to analyze the phylogenetic characterization of TBPs detected in these ticks. The adult *Rh. microplus* (*n* = 516) feeding on cattle were collected. The pooled DNA samples of these ticks were evaluated using metagenomic next-generation sequencing (mNGS) and then TBPs in individual ticks were identified using genus- or group-specific nested polymerase chain reaction (PCR) combined with DNA sequencing assay. As a result, *Candidatus Rickettsia jingxinensis* (24.61%, 127/516), *Anaplasma marginale* (13.18%, 68/516), *Coxiella burnetii* (3.10%, 16/516), and *Coxiella-*like endosymbiont (CLE) (8.33%, 43/516) were detected. The dual coinfection with *Ca. R. jingxinensis* and *A. marginale* and the triple coinfection with *Ca. R. jingxinensis*, *A. marginale*, and CLE were most frequent and detected in 3.68% (19/516) and 3.10% (16/516) of these ticks, respectively. The results provide insight into the diversity of TBPs and their coinfections in *Rh. microplus* in Yunnan province of China, reporting for the first time that *C. burnetii* had been found in *Rh. microplus* in China. Multilocus variable number tandem repeat analysis with 6 loci (MLVA-6) discriminated the *C. burnetii* detected in *Rh. microplus* in Yunnan into MLVA genotype 1, which is closely related to previously described genotypes found primarily in tick and human samples from different regions of the globe, indicating a potential public health threat posed by *C. burnetii* in *Rh. microplus* in Yunnan.

## Introduction

Ticks are distributed widely across the world, and approximately 10% of the currently known 867 tick species act as arthropod vectors in the transmission of human and animal pathogens (Zhang et al., 2019; Yang et al., 2021). Likely due to human movement into tick habitats combined with climate changes, tick-borne diseases are increasing in prevalence and present an increasing global concern (Ostfeld and Brunner, 2015). As tick-borne diseases become more prevalent, the likelihood of coinfection with more than one tick-borne pathogen (TBP) in ticks is increasing and such coinfections have important repercussions on human or animal health which can alter clinical presentation, disease severity, and treatment response in tick-borne diseases (Vaumourin et al., 2015) and may play a role in incidence, distribution, and possible control of tick-borne diseases (Andersson et al., 2017).

Risk of human infection is contingent on the geographic distribution of the tick species as well as the prevalence of TBPs carried by ticks in a given region (Wilson and Elston, 2018). *Rhipicephalus microplus* is a common vector for the transmission of a great variety of microorganisms including bacteria, viruses, protozoa, fungi, or toxins. Among the TBPs in *Rh. microplus*, *Coxiella burnetii*, an obligate intracellular bacterium and the pathogenic agent of Q fever, is worldwide distributed. Q fever is typically an acute febrile illness with non-specific clinical signs in humans, but Q fever may manifest in human as an acute hepatitis and pneumonia or as chronic diseases that are seen in severe cases or life-threatening diseases such as valvular endocarditis (Sulyok et al., 2014).

*Rhipicephalus microplus* have been proved to be one of six most frequently reported tick species in China and mostly distributed in the southeast part of China (Zhang et al., 2019). *Anaplasma* spp., *Theileria* spp., *Ehrlichia* spp., *Hepatozoon canis*, and the viral community (Wen et al., 2003; Chen et al., 2014; Guo et al., 2019b; Li et al., 2020a; Shi et al., 2021) have been reported to be found in *Rh. microplus* in the region, indicating a high risk of exposure to these pathogens for humans and animals here.

Yunnan province is located in the southwestern part of China with wide distribution of *Rh. microplus*; it is of great importance for epidemiologists and physicians to be aware of *Rh. microplus* here for evaluating their potential for spread of the tick-borne diseases. In the present study, we investigated the potential TBPs in *Rh. microplus* collected from cattle in Yunnan. The results in the present study might provide a better understanding of TBPs carried by *Rh. microplus* in Yunnan, thereby strengthening programs to prevent and control the potential infections caused by these TBPs.

## Materials and Methods

### Tick Collection and Identification

**FIGURE 1:**
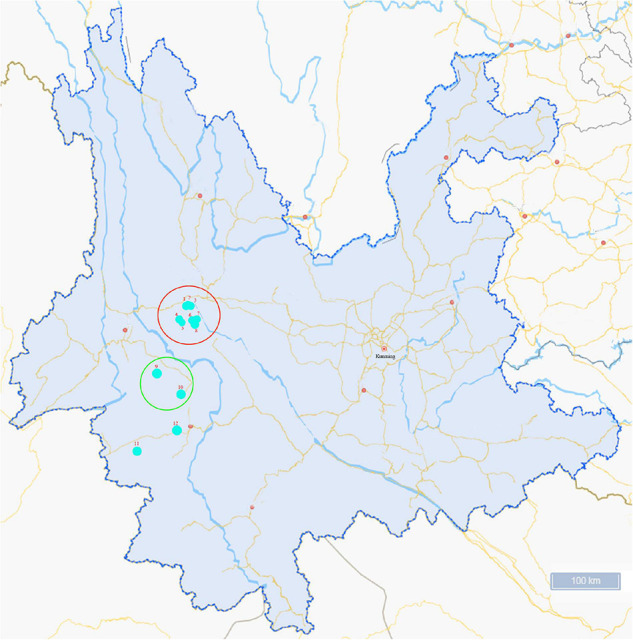
Map of the sampling sites in Yunnan Province, China. Ticks collected were fed on cattle in Lincang city (sampling sites 1–8) and Weishan city (sampling sites 9–12). The colored circles indicate the two cities where the ticks were collected and the dots indicate the sampling regions in this study.

An investigation was conducted from June to August in 2020, and ticks collected were fed on cattle in Lincang city and Weishan city in Yunnan province (Figure 1). Tick species were identified based on morphological characterization and by molecular biology methods based on the sequences of species-specific *16S rRNA* and mitochondrial cytochrome c oxidase I (*COI*) genes, as previously described (Chitimia et al., 2010). Following identification, the ticks were stored at −80°C for further analysis.

### DNA Extraction

To remove environmental contaminants, ticks were individually surface sterilized by 75% ethanol twice, followed by phosphate-buffered saline (PBS) twice. Ticks were then individually homogenized in 300 μl of PBS using the MagNA Lyser Green Beads (Roche, Mannheim, Germany), and DNA extraction was performed on 200 μl of each tick homogenate using QIAamp^®^ Fast DNA Tissue Kit (Qiagen, Düsseldorf, Germany) according to the manufacturer’s instructions. The extracted genomic DNA was dissolved in 100 μl ultrapure water and stored at −20°C for further analysis. Individual DNA samples were mixed in an equal volume (10 μl) to prepare pooled DNA samples for full microbial genome sequencing using mNGS.

### Metagenomic Profiling

All pooled DNA samples were paired-end sequenced on the Illumina HiSeq platform (insert size 350 bp, read length 150 bp) by The Beijing Genomics Institute (BGI) (Beijing, China), and the depth of sequencing for all pooled DNA samples was 10×. The reads with more than 40-nt low-quality bases (quality value ≤38) were removed. Meanwhile, the reads with more than 10-nt “N” bases were filtered out of the datasets. Lastly, the reads overlapping more than 15-nt bases with the adapters were removed. Reads that aligned to tick genes were also removed using Bowtie 2 (v2.2.4) (Karlsson et al., 2012, 2013). Accordingly, the high-quality data were obtained.

Then, the high-quality reads were mapped against scaffolds using SOAPdenovo (v2.04) (Luo et al., 2012). The unused reads from each sample were then assembled. The scaffolds were broken at N into the scaftigs (Nielsen et al., 2014), and the scaftigs with the length of ≥500 nt were used for further analysis (Li et al., 2014). Open reading frames (ORFs) in the scaftigs (≥500 bp) were predicted by MetaGeneMark (v2.10) (Karlsson et al., 2012; Qin et al., 2014). A non-redundant gene catalog was obtained after processing by using the CD-HIT (v4.5.8) (Li and Godzik, 2006; Fu et al., 2012). To determine the gene abundances, the reads were realigned with the gene catalog using Bowtie 2. Relative abundance of genes was calculated based on the number of reads mapped to the genes and the length of the genes, as previously described (Cotillard et al., 2013; Le Chatelier et al., 2013; Villar et al., 2015).

To access the taxonomic assignments of genes, genes were aligned to the integrated NR database (Version: 2018-01-02) of NCBI using DIAMOND (v0.9.9) (Buchfink et al., 2015). Then, the taxonomical level of each gene was determined by using the lowest common ancestor (LCA)-based algorithm implemented in MEGAN (Huson et al., 2007). The results containing the number of genes and the abundance information of each sample and the relative abundances of each taxonomic group were calculated by adding the relative abundances of genes annotated to the same feature (Karlsson et al., 2012; Li et al., 2014; Feng et al., 2015).

### Polymerase Chain Reaction

Based on the results of mNGS, genus/group-specific PCR was performed to confirm the presence of TBPs in individual ticks. For nested PCR, 1 μl of each individual DNA sample was used as template for the first round and 1 μl of the primary PCR production was used as template for the second round. The target genes and specific primers for the spotted fever group rickettsia (SFGR; Cheng et al., 2016), *Anaplasma* spp. and *Ehrlichia* spp. (Qin et al., 2018), and *Coxiella* spp. (Duron et al., 2015) are listed in Table 1. All PCR amplifications were carried out using the PrimeSTAR^®^ HS (Premix) (TaKaRa, Beijing, China) and performed on the PCR System 9700 (Applied Biosystems, GeneAmp^®^, United States). Amplified products were then electrophoresed in 1.5% agarose gel, and the positive amplicons were sent to TSINGKE Biological Technology (Beijing, China) for sequencing.

**TABLE 1:** Target genes and primer sequences used for nested PCR.

**Pathogen**	**Target gene**	**Primer name**	**Sequence (5′–3′)**	**Tm (T/C)**
SFGR	*gltA*	CS2d	ATGACCAATGAAAATAATAAT	50
		CSEndr	CTTATACTCTCTATGTACA	
		RpCS.877p	GGGGACCTGCTCACGGCGG	48
		RpCS.1258n	ATTGCAAAAAGTACAGTGAACA	
*Anaplasma* spp.	*16S rRNA*	Eh-out1	TTGAGAGTTTGATCCTGGCTCAGAACG	55
*Ehrlichia* spp.		Eh-out2	CACCTCTACACTAGGAATTCCGCTATC	
		Eh-gs1	GTAATAACTGTATAATCCCTG	55
		Eh-gs2	GTACCGTCATTATCTTCCCTA	
*Coxiella* spp.	*16S rRNA*	Cox16SF1	CGTAGGAATCTACCTTRTAGWGG	55
		Cox16SR2	GCCTACCCGCTTCTGGTACAATT	
		Cox16SF1	CGTAGGAATCTACCTTRTAGWGG	55
		Cox16SR1	ACTYYCCAACAGCTAGTTCTCA	

### Phylogenetic Analysis

The obtained DNA sequences were compared with those available in GenBank using the National Center for Biotechnology Information (NCBI; Bethesda, MD, United States) Basic Local Alignment Search Tool (BLAST) search engine^1^, and multiple-sequence alignment was performed using the ClustalW multiple alignment tool with the default parameters in the MEGA X. The phylogenetic analysis of *gltA* for SFGR, *16S rRNA* for *Anaplasma* spp., or *16S rRNA* for *Coxiella* spp. was performed using the maximum likelihood method based in MEGA X. Bootstrap values were estimated for 1,000 replicates (Hall, 2013; Kumar et al., 2016).

### Multilocus Variable Number Tandem Repeat Analysis

Multilocus variable number tandem repeat analysis was performed in PCR targeting six highly variable loci, including ms23, ms24, ms27, ms28, ms33, and ms34 (Arricau-Bouvery et al., 2006). The forward and reverse primer sequences and PCR conditions were applied as described previously (Klaassen et al., 2009; Tilburg et al., 2012; Gonzalez-Barrio et al., 2016). A *C. burnetii* strain (Nine Mile) which was considered 9–27–4–6–9–5 for loci ms23–ms24–ms27–ms28–ms33–ms34 was used as the reference for normalization and for comparing the MLVA profiles obtained. The MLVA pattern of the isolates was compared in the database of MLVABank^2^ to check similarities with other isolates. Clustering of obtained MLVA profiles was performed with BioNumerics v.7.6 software (Applied Maths, Belgium).

## Results

### Taxonomic Classification

All adult hard ticks collected were identified as *Rh. microplus* (*n* = 516) based on morphological identifications and confirmed by species-specific PCR and sequencing assay. Fifteen pools of *Rh. microplus* DNA samples were finally analyzed using mNGS. Sequencing yielded between 6,166 and 7,273 million reads per pool, while all were of high quality (Clean_Q20 > 96%) (Supplementary Table 1).

**FIGURE 2:**
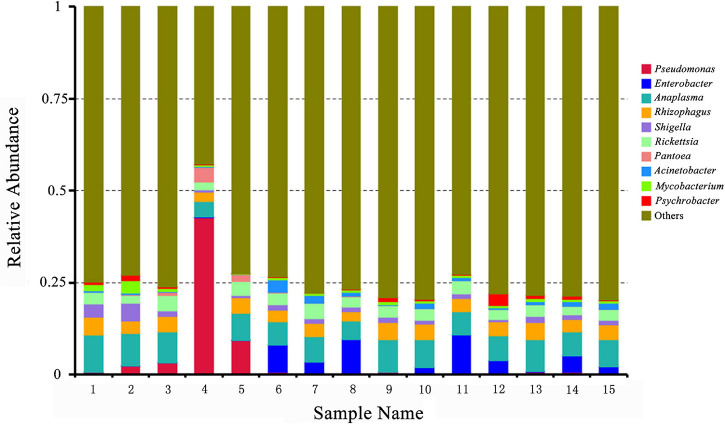
Relative abundances of potential top 10 pathogens at the genus level in pooled *Rhipicephalus microplus* samples. The *x*-axis indicates pooled DNA sample names of *Rh. microplus* for metagenomic next-generation sequencing; the *y*-axis indicates relative abundance. “Others” indicates the sum of the relative abundance of all genera except the top 10.

Then, the presence of *Rickettsia* and *Anaplasma* in the pooled tick samples was identified by the taxonomic profiles at the genus level (Supplementary Table 2) and the 10 most abundant bacterial genera in pooled tick samples are as shown in Figure 2. *Rickettsia* spp. and *Anaplasma* spp. were abundant in all the sample pools. In addition, *Pseudomonas* spp. was most abundant in pool 4.

### Tick-Borne Pathogen Identification

By mNGS, the important pathogenic bacterial genera *Rickettsia* and *Anaplasma* were found in the pooled tick samples, and thus each tick sample was analyzed by the genus/group-specific PCR combined with sequencing in order to identify TBPs carried by it. In addition, *Coxiella* spp. was commonly found in ticks, and thus each tick sample was also analyzed by *Coxiella*-specific PCR.

As a result, 34.69% (179/516) of ticks were tested positive for at least one of the four pathogens/endosymbionts [*Candidatus Rickettsia jingxinensis*, *Anaplasma marginale*, *C. burnetii*, and *Coxiella*-like endosymbiont (CLE)] (Table 2). *Ca. R. jingxinensis* was the highest prevalence in these ticks (24.61%, 127/516), while the prevalence of *A. marginale* and *C. burnetii* were 13.18 and 3.10% in these ticks, respectively (Table 2).

**TABLE 2:** Prevalence of tick-borne pathogens in individual tick.

**Pathogen**	**Number of individual tick positive for single and coinfections**
	***Rhipicephalus microplus* (*n* = 516)**
**Single**	
*Candidatus Rickettsia jingxinensis*	74 (14.34%)
*Anaplasma marginale*	27 (5.23%)
*Coxiella burnetii*	14 (2.71%)
*Coxiella*-like endosymbiont	5 (0.97%)
**Double**	
*Ca. R. jingxinensis*, *A. marginale*	19 (3.68%)
*A. marginale*, *C. burnetii*	2 (0.39%)
*A. marginale*, *Coxiella*-like endosymbiont	4 (0.78%)
*Ca. R. jingxinensis*, *Coxiella*-like endosymbiont	18 (3.49%)
**Triple**	
*Ca. R. jingxinensis*, *A. marginale*, *Coxiella*-like endosymbiont	16 (3.10%)
Total	179 (34.69%)

### Coinfections

In 179 TBP-positive ticks in the present study, 59 ticks (32.96%) were found to be coinfected with more than one TBP identified (Table 2). The dual coinfections with *Ca. R. jingxinensis* and *A. marginale* (8.33%, 43/516) were most frequent in *Rh. microplus*, while the dual coinfections with *A. marginale* and *C. burnetii*, the dual coinfection with *A. marginale* and CLE, and the dual coinfection with *Ca. R. jingxinensis* and CLE were detected in 0.39% (2/516), 0.78% (4/516), and 3.49% (18/516) of these ticks, respectively. The triple coinfections with *Ca. R. jingxinensis*, *A. marginale*, and CLE were detected in 3.10% (16/516) of these ticks (Table 2).

### Phylogenetic Analysis

**FIGURE 3:**
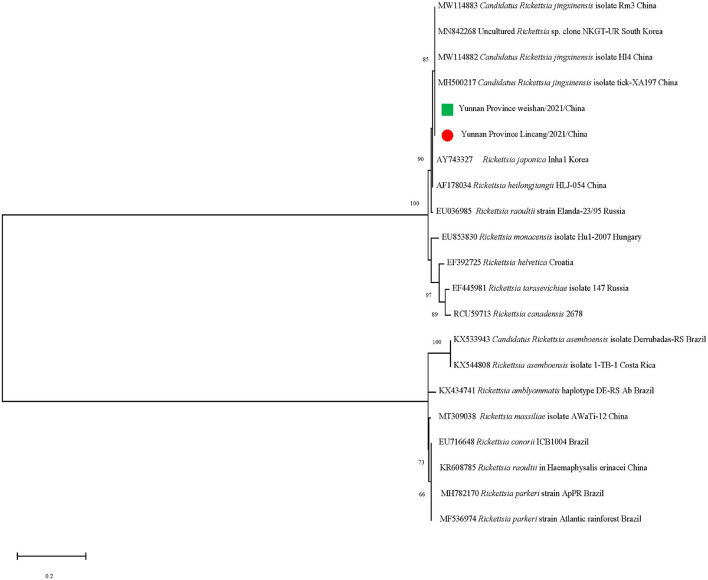
Phylogenetic tree of *Candidatus Rickettsia jingxinensis* in ticks based on partial *gltA* gene sequence similarity. The sequences obtained in this study are indicated with a colored dot. Sequences were aligned using the MEGA X (Version 10.2.5) software package. Phylogenetic analysis was performed by the maximum likelihood method, and bootstrap values were estimated for 1,000 replicates.

**FIGURE 4:**
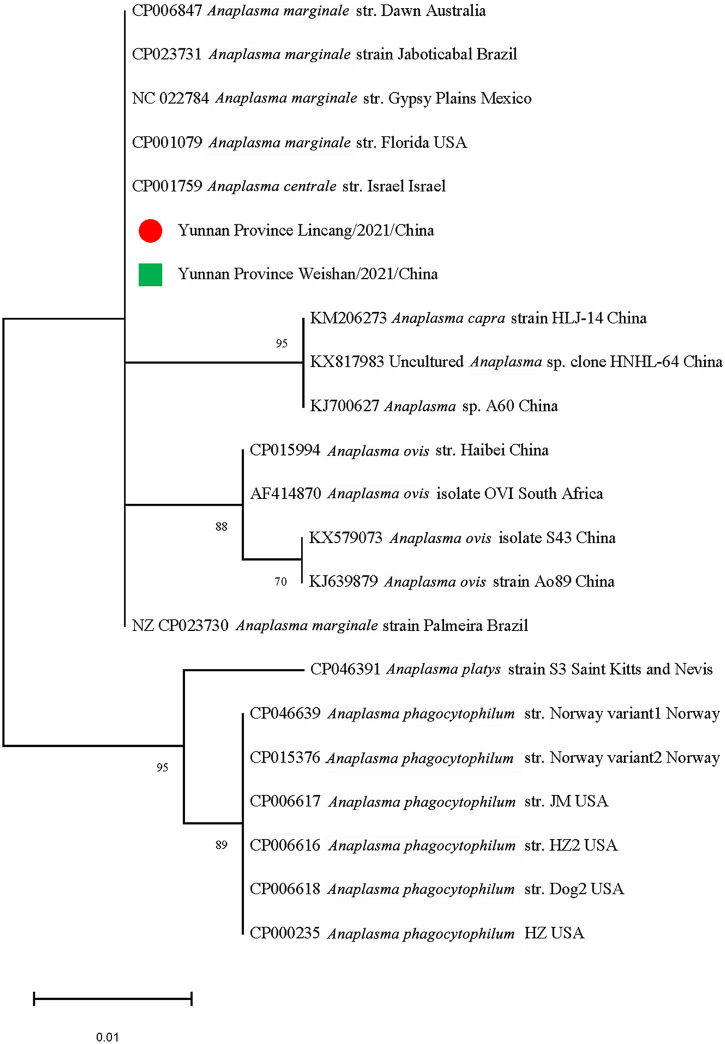
Phylogenetic tree of *Anaplasma marginale* in ticks based on partial *16S rRNA* gene sequence similarity. The sequences obtained in this study are indicated with a colored dot. Sequences were aligned using the MEGA X (Version 10.2.5) software package. Phylogenetic analysis was performed by the maximum likelihood method, and bootstrap values were estimated for 1,000 replicates.

**FIGURE 5:**
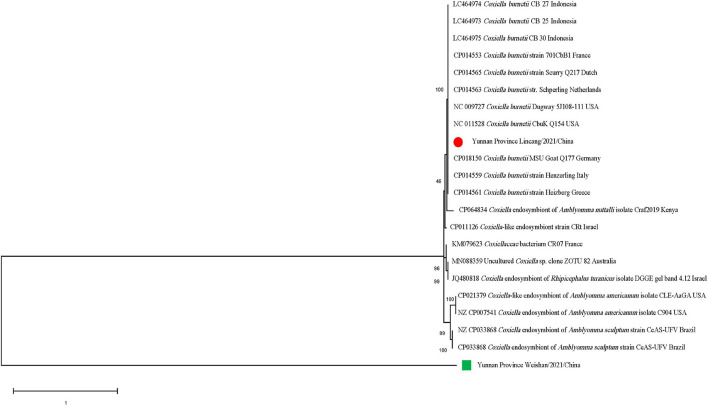
Phylogenetic tree of *Coxiella* spp. in ticks based on partial *16S rRNA* gene sequence similarity. The sequences obtained in this study are indicated with a colored dot. Sequences were aligned using the MEGA X (Version 10.2.5) software package. Phylogenetic analysis was performed by the maximum likelihood method and bootstrap values were estimated for 1,000 replicates.

The *gltA* sequences of *Ca. R. jingxinensis* and *16S rRNA* of *A. marginale* and *C. burnetii* in this study were 100% identical to those in GenBank, while CLE detected in the *Rh. microplus* collected in Weishan showed 97.57–99.20% nucleotide sequence identity to the known CLE strains and was most similar to the CLE strain (JQ480818.1) detected in *Rhipicephalus turanicus* in the *16S rRNA* comparison. By phylogenetic analysis, *Ca. R. jingxinensis* detected in the present study were placed in a clade with *Ca. R. jingxinensis* (MH500217, MW114882, and MW114883) and an uncultured *Rickettsia* sp. clone NKGT-UR (MN842268) (Figure 3). The *A. marginale* identified was shown to be clustered with *A. marginale* (CP006847, CP023731, NC022784, and CP001079) and *Anaplasma centrale* str. Israel (CP001759) (Figure 4). *C. burnetii* was most close to *C. burnetii* CbuK (NC011528) and *C. burnetii* Dugway 5J108-111 (NC009727), while CLE detected in the present study was placed in a separated clade with the known CLE strains (Figure 5).

### Multilocus Variable Number Tandem Repeat Analysis Typing

**FIGURE 6:**
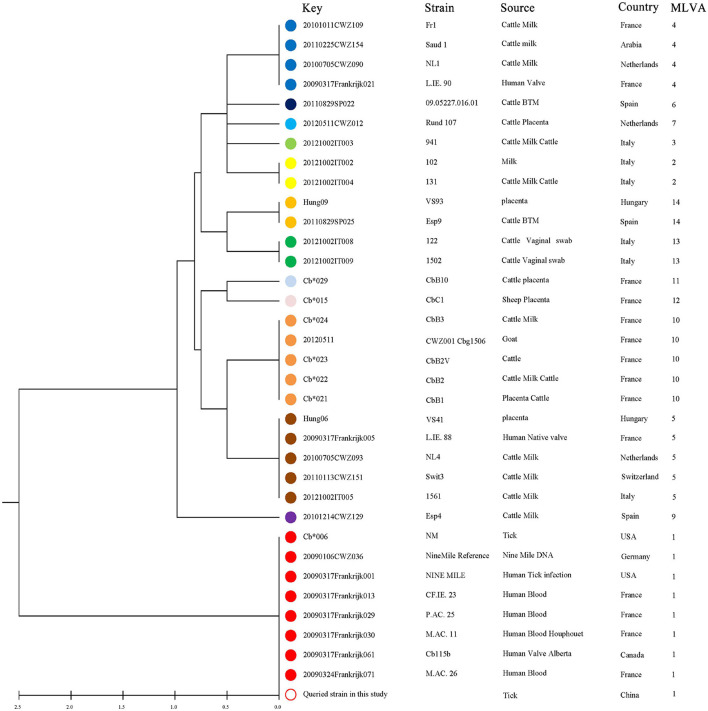
UPGMA cluster analysis of *Coxiella burnetii* genotypes using a six-multiple locus variable number tandem repeat analysis. All 34 selected samples are based on the MLVA-6 database (http://mlva.i2bc.paris-saclay.fr/mlvav4/genotyping/). The reference strain included in the tree is Nine Mile RSA493. Strain, source, geographical origin, and MLVA-6 type are indicated. The same genotype was coded with the same color. The hollow dots indicate the sample genotype obtained in this study.

**FIGURE 7:**
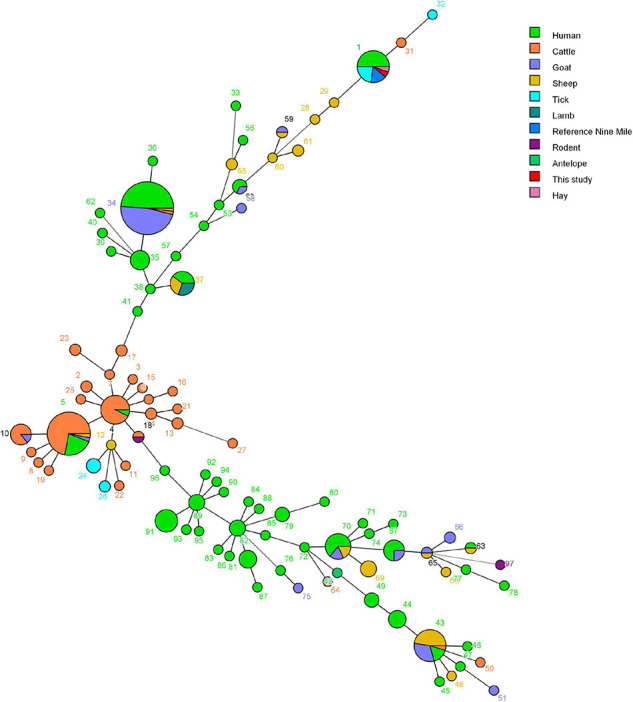
Minimum spanning tree of *Coxiella burnetii* strains using a six-multiple locus variable number tandem repeat analysis. The obtained MLVA genotypes identified in this study and data of 304 strains based on the MLVA-6 database (http://mlva.i2bc.paris-saclay.fr/mlvav4/genotyping/) were used. Each circle represents a unique genotype (1–97). The minimum spanning tree of the analyzed isolates provides information on the proportion of hosts of each identified genotype (see color index), and the size of the pie charts represents the number of isolates of the corresponding genotype.

In total, 14 tick DNA samples positive for *C. burnetii* and 10 DNA samples were characterized by a complete MLVA analysis, and not all *C. burnetii* DNA-positive samples could be characterized probably due to the poor quality and quantity of DNA. The allele codes of the identified MLVA type were 9–27–4–6–9–5 for loci ms23–ms24–ms27–ms28–ms33–ms34 and recognized as MLVA genotype 1, suggesting that these 10 strains belong to the same genotype and were closely related to tick *Coxiella* strains isolated from United States and human *Coxiella* strains isolated from United States, France, and Canada (Figure 6). A minimum spanning tree based on host origin of the MLVA analysis showed that the *C. burnetii* strains detected in the present study were clustered to the previously described genotypes found primarily in ticks and patients of Q fever from different regions of the globe (Figure 7).

## Discussion

Although a variety of pathogens have been identified in ticks, the single infection and coinfection with multiple pathogens in *Rh. microplus* have rarely been investigated in China. In this study, we applied mNGS combined with nested PCR to survey TBPs in *Rh. microplus* feeding on cattle in Yunnan province and to analyze the phylogenetic characterization of TBPs detected in these ticks.

The pooled DNA samples of *Rh. microplus* collected were assayed by mNGS. Although each tick was surface sterilized and ultrapure water, sterile tubes, and filter tips were used and all operations were carried out in a biological safety cabinet during DNA extraction, bacteria like *Pseudomonas*, *Enterobacter*, and *Rhizophagus* were abundant in the pooled DNA samples of *Rh. microplus* when analyzed using mNGS. Pathogenic, environmental, and skin-associated bacteria have also been reported as highly abundant (Greay et al., 2018), and it is one of the possibilities that bacteria like *Pseudomonas* were predominant in the present study. *Rickettsia* spp. and *Anaplasm*a spp. were also revealed at the genus level in pooled DNA samples. Moreover, *Ca. R. jingxinensis*, *A. marginale*, *C. burnetii*, and CLE were further identified in individual tick DNA by sequencing of PCR-amplified DNA fragments. *Coxiella* spp. was not identified in the pooled DNA sample *via* mNGS, and one of the possibilities is the degradation of nucleic acids during sequencing.

Spotted fever group rickettsias are devastating human infections, and no licensed vaccine is available (Liu et al., 2021). More than 20 species of *Rickettsia* are associated with SFGR, of which 16 are considered as human pathogens (Cohen et al., 2021). *Ca. R. jingxinensis*, one uncultured SFGR species, was identified in 24.61% of these ticks. The sequence of *Ca. R. jingxinensis* was first described in *Haemaphysalis longicornis* in Japan (Ishikura et al., 2003), and its presence in *Ha. longicornis* or *Rh. microplus* has been reported in China (Zou et al., 2011; Dong et al., 2014) and then was named based on its geographical origin in 2016 (Liu et al., 2016). Many *Ca. R. jingxinensis*-specific DNA sequences have been deposited in GenBank. Of these, a *gltA* sequence (KU853023) was recovered from a patient, suggesting its potential pathogenicity to humans (Guo et al., 2019a). Our analysis showed that the *gltA* of *Ca. R. jingxinensis* in *Rh. microplus* was 100% identical to that of *Candidatus Rickettsia longicornii*, suggesting that the two organisms should be recognized as one species, which is consistent with the previous report (Jiang et al., 2018). These results revealed that *Ca. R. jingxinensis* is widely distributed in China even in the world and its pathogenicity remains to be determined.

*Anaplasma marginale*, the causative agent of bovine anaplasmosis, can be transmitted by at least 20 species of ticks mainly in the genera *Dermacentor* and *Rhipicephalus* (Duan et al., 2020). The infection rate of *A. marginale* is determined by the level of rickettsemia in the host and the ability to infect the midgut of tick vector and undergo successful biological replication (Ueti et al., 2007). *A. marginale* has been detected in ticks from Ningxia, Hubei, and Henan provinces and the Qinghai-Tibet Plateau in China (Lu et al., 2017; Cui et al., 2018; Han et al., 2019; Duan et al., 2020). The presence of *A. marginale* has been detected in 13.18% of *Rh. microplus* in the present study, suggesting its wider geographical distribution in China.

*Coxiella burnetii* is the causative agent of Q fever, and this bacterium is highly infectious and classified as a category B biological weapon (Madariaga et al., 2003). In the 1950s, Q fever was first reported in China and then *C. burnetii* has been isolated from patients, livestock, wild mice, and ticks (Ni et al., 2020). The sporadic human Q fever cases and several small outbreaks of Q fever that occurred in leather factories or goat/sheep farms were reported in China (Yu, 2000). In 2018–2019, an epidemic of human Q fever in Zhuhai city of China was determined by mNGS (Huang et al., 2021). In the present study, *C. burnetii* were detected in 3.10% of *Rh. microplus* (14/516), while the previous reports show that it was detected in 12.50% of *Dermacentor nuttalli* (7/56), 2.79% of *Dermacentor silvarum* (11/394), 14.75% of *Dermacentor niveus* (9/61), and 22.65% of *Hyalomma asiaticum* (41/181) in China (El-Mahallawy et al., 2015; Li et al., 2020b,c; Ni et al., 2020), strongly demonstrating its wide distribution in China. *C. burnetii* has been detected in *Rh. microplus* in the Philippines, Thailand, and Mali before (Muramatsu et al., 2014; Diarra et al., 2017; Galay et al., 2020), and this is the first time that *C. burnetii* has been detected in *Rh. microplus* in China.

Genotyping *C. burnetii* from wildlife will help in tracing back clinical cases in humans directly exposed to wildlife. MLVA is nowadays the first-choice method to compare *C. burnetii* genotypes due to its powerful method to type *C. burnetii* from a diversity of hosts and geographic origins (Klaassen et al., 2009; Roest et al., 2011). In the present study, *C. burnetii* genotypes 1 were obtained using the MLVA-6-marker and genotype 1 was mainly found in strains from both patients and ticks. *C. burnetii* genotypes from ticks in the present study clustered mainly with *C. burnetii* genotypes from human Q fever cases in France, Canada, and United States (Figures 6, 7), suggesting that these strains identified in the ticks in Yunnan province were phylogenetically closely related to the strains from ticks and patients in different regions of the world. Interestingly, Q fever in humans in Yunnan province where our samples were collected has been reported (Fang et al., 2015). *C. burnetii* is a zoonotic pathogen transmitted from infected vertebrate animals to humans *via* contaminated aerosols (Graves et al., 2016). Although direct transmission of *C. burnetii* to humans through ticks has never been properly documented (Pacheco et al., 2013), tick bite cannot be ruled out and a previous report has suggested that Q fever may have been transmitted by tick bite (Beaman and Hung, 1989), and ticks may play a critical role in the transmission of *C. burnetii* among vertebrate animals (both domestic and native). To the best of our knowledge, this is the first time that *C. burnetii* found in China has been genotyped using MLVA.

A common characteristic of the various tick species is the presence of bacterial endosymbionts, typically bacterial members of *Coxiella*, *Rickettsia*, and *Francisella* genera, some of which are closely related to vertebrate pathogens (Tsementzi et al., 2018). CLEs are uncultured and relatively common in the microbiota of various tick species around the world and affect the development, nutrition, chemical defense, or reproduction of the hosts (Ben-Yosef et al., 2020). CLEs form multiple subclusters in the cluster of the genus *Coxiella* in phylogenetic analysis (Duron et al., 2015). CLE has not been reported in *Rh. microplus* before, and in the present study, CLEs were detected in 8.33% of *Rh. microplus*, similar to the prevalence level of CLE in *Rhipicephalus sanguineus* in North America and Europe (Ueti et al., 2007). More importantly, CLE detected in *Rh. microplus* collected from Weishan shared 97.57–99.20% of *16S rRNA* sequence identity with the known CLE strains, forming a separated clade in phylogenetic analysis.

## Conclusion

This study provides a better understanding of TBPs in *Rh. microplus* in Yunnan and the presence of TBP coinfections in *Rh. microplus*, reporting for the first time that *C. burnetii* had been found in *Rh. microplus* in China. MLVA data-based phylogenetic analysis showed that the *C. burnetii* strains detected in *Rh. microplus* in Yunnan belonged to MLVA genotype 1, which is closely related to previously described genotypes found primarily in ticks and patients from different regions in the world, and suggesting a potential public health threat to the people living close to the natural foci of *C. burnetii*. It is important to plan future surveys, applying molecular methods for investigation of these TBPs in livestocks and farmers closely related to *Rh. microplus* in Yunnan province of China.

## Data Availability Statement

The datasets presented in this study can be found in online repositories. The names of the repository/repositories and accession number(s) can be found below: https://www.ebi.ac.uk/, ERS6645636.

## Author Contributions

JJ and XX conceived and designed the study and drafted the manuscript. JZ, PH, YS, and QY performed the sample collection, tick species identification, and laboratory work. JJ, XO, and YY performed the experimental data analysis. BW and QY edited the manuscript. All authors contributed to the article and approved the submitted version.

## Conflict of Interest

The authors declare that the research was conducted in the absence of any commercial or financial relationships that could be construed as a potential conflict of interest.

## Publisher’s Note

All claims expressed in this article are solely those of the authors and do not necessarily represent those of their affiliated organizations, or those of the publisher, the editors and the reviewers. Any product that may be evaluated in this article, or claim that may be made by its manufacturer, is not guaranteed or endorsed by the publisher.

## Footnotes

1
http://blast.ncbi.nlm.nih.gov/blast.cgi

2
http://mlva.u-psud.fr/mlvav4/genotyping/
